# Evaluation of Ga-DOTA-(D-Asp)_n_ as bone imaging agents: D-aspartic acid peptides as carriers to bone

**DOI:** 10.1038/s41598-017-14149-7

**Published:** 2017-10-25

**Authors:** Kazuma Ogawa, Atsushi Ishizaki, Kenichiro Takai, Yoji Kitamura, Akira Makino, Takashi Kozaka, Yasushi Kiyono, Kazuhiro Shiba, Akira Odani

**Affiliations:** 1 Graduate School of Medical Sciences, Kanazawa University, Kanazawa, Japan; 2 Institute for Frontier Science Initiative, Kanazawa University, Kanazawa, Japan; 3 Advanced Science Research Center, Kanazawa University, Kanazawa, Japan; 4 Biomedical Imaging Research Center, University of Fukui, Eiheiji, Japan

## Abstract

^67^Ga-DOTA-(L-Asp)_11_ and ^67^Ga-DOTA-(L-Asp)_14_, which have been developed as bone imaging agents, showed a high accumulation in bone and a rapid blood clearance in mice. However, peptides composed of D-amino acids are more stable *in vivo* than those composed of their L-equivalents. In this study, ^67^Ga-DOTA-(D-Asp)_n_ (n = 2, 5, 8, 11, or 14) were synthesized using the Fmoc-based solid-phase methodology and evaluated. In hydroxyapatite binding assay, binding of ^67^Ga-DOTA-(D-Asp)_n_ tended to increase with increasing length of the amino acid chain. ^67^Ga-DOTA-(D-Asp)_11_ and ^67^Ga-DOTA-(D-Asp)_14_ caused a high accumulation of radioactivity in the bones of the mice. However, the results for ^67^Ga-DOTA-(D-Asp)_n_ and ^67^Ga-DOTA-(L-Asp)_n_ were comparable. In urine analyses, the proportion of intact complex after injection of ^67^Ga-DOTA-(D-Asp)_14_ was significantly higher than that of ^67^Ga-DOTA-(L-Asp)_14_. Although ^67^Ga-DOTA-(D-Asp)_14_ was more stable than ^67^Ga-DOTA-(L-Asp)_14_, the properties of ^67^Ga-DOTA-(D-Asp)_n_ and ^67^Ga-DOTA-(L-Asp)_n_ as bone imaging agents may be comparable.

## Introduction

Recently, the performance of X-ray computed tomography (CT) and magnetic resonance imaging (MRI) method has been greatly improved, particularly in terms of their spatial resolution and technology for reconstructing the acquired images. Nuclear medicine imaging has been considered to be the most sensitive approach for diagnosing bone disorders such as bone metastases due to its ability to enable the early detection of abnormalities, namely, visualization of lesion sites before anatomical changes. For a long time, ^99m^Tc-methylenediphosphonate (^99m^Tc-MDP) and ^99m^Tc-hydroxymethylenediphosphonate (^99m^Tc-HMDP) have been widely used in bone imaging^1–5^. Because ^99m^Tc has the convenient physical characteristics [moderate half-life (6.01 h) for clinical use, a generator-produced radionuclide, and appropriate gamma ray energy for imaging] and imaging methods using conventional gamma cameras are simple. ^99m^Tc-MDP and ^99m^Tc-HMDP are complexes of ^99m^Tc with bisphosphonate analogs having high affinity for bone since the phosphate groups in the bisphosphonate can be coordinated with calcium in hydroxyapatite crystals in bone.

The use of [^18^F]NaF for bone imaging was initially reported by Blau *et al*. in 1962^6^ and approved by the US Food and Drug Administration in 1972. [^18^F]NaF accumulates at a high level in bone because of chemisorption with the exchange of fluoride anions with the hydroxyl groups in hydroxyapatite [Ca_10_(PO_4_)_6_(OH)_2_]. However, [^18^F]NaF had not been widely used due to its limited availability and high cost, but it has recently been reevaluated. The images obtained using clinical positron emission tomography (PET) generally have high spatial resolution and PET/CT scanners have become widely available commercially. Although Even-Sapir *et al*. reported that [^18^F]NaF PET imaging is significantly more sensitive than ^99m^Tc-MDP planar and ^99m^Tc-MDP single photon emission computed tomography (SPECT) imaging^7^, the problems of limited availability and the high cost of cyclotrons have remained unresolved.

In recent years, ^68^Ga (*T*_1/2_ = 68 min) has drawn substantial attention as a positron emission radionuclide for clinical PET because of its attractive radiophysical properties, such as reasonable half-life for clinical use; it has particularly been used as a generator-produced radionuclide. ^68^Ga-PET does not require an on-site cyclotron because ^68^Ga can be eluted from the generator on demand. Moreover, as the parent nuclide, ^68^Ge (*T*_1/2_ = 271 days) has a long half-life, a generator could be used for a long period. Therefore, the demand for ^68^Ga-labeled compounds for the diagnosis of bone disorders, such as bone metastases, has increased. Some new radiogallium-labeled complexes for bone imaging have been developed in recent years^8–14^. Bisphosphonate analogs are used as carriers in these radiogallium-labeled complexes. For example, Fellner *et al*. reported that ^68^Ga-DOTA-conjugated bisphosphonate, ^68^Ga-BPAMD, showed high uptake in osteoblastic metastatic lesions in a first human PET study^15^. In addition, Suzuki *et al*. reported that ^68^Ga-NOTA-conjugated bisphosphonate, ^68^Ga-NOTA-BP, showed high bone affinity and rapid blood clearance in animal experiments^10^.

**Figure 1:**
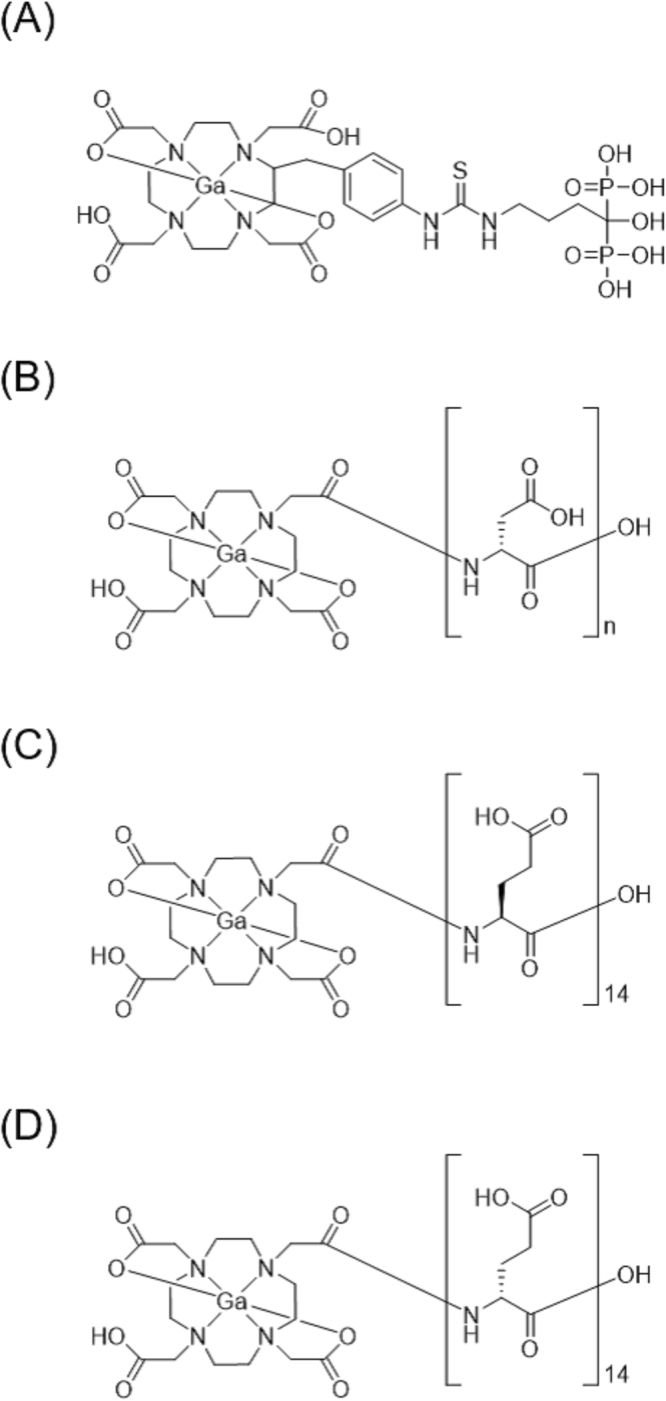
Chemical structures of (**A**) Ga-DOTA-Bn-SCN-HBP, (**B**) Ga-DOTA-(D-Asp)_n_ (n = 2, 5, 8, 11, or 14), (**C**) Ga-DOTA-(L-Glu)_14_, and (**D**) Ga-DOTA-(D-Glu)_14_.

The acidic amino acid peptides (poly-glutamic and poly-aspartic acids) also have a high affinity for hydroxyapatite because side-chain carboxyl groups in the acidic amino acid peptides can be coordinated with calcium in hydroxyapatite, and could become carriers delivering drugs to bone^16–18^. Recently, 1,4,7,10-tetraazacyclododecane-1,4,7,10-tetraacetic acid (DOTA) has been used as a chelating site, and Ga-DOTA-conjugated aspartic acid peptides [Ga-DOTA-(L-Asp)_n_], with varying peptide lengths (n = 2, 5, 8, 11, or 14), have been developed and evaluated using the easy-to-handle radioisotope ^67^Ga, which has a longer half-life (3.3 days), rather than ^68^Ga^19^. ^67^Ga-DOTA-(L-Asp)_11_ and ^67^Ga-DOTA-(L-Asp)_14_ show high affinity for hydroxyapatite, high accumulation in bone, and rapid blood clearance in biodistribution experiments in normal mice. Accordingly, the bone/blood ratios of ^67^Ga-DOTA-(L-Asp)_11_ and ^67^Ga-DOTA-(L-Asp)_14_ are comparable to those of ^99m^Tc-HMDP and ^67^Ga-DOTA-Bn-SCN-HBP (Fig. 1A), a Ga-DOTA-conjugated bisphosphonate, which was developed and evaluated in our previous study^11^. In these Ga-DOTA-conjugated aspartic acid peptide compounds, L-aspartic acid is used as the only component of the peptides. However, the peptides composed of D-amino acids could be more stable *in vivo* than the peptides built with L-amino acids because they are not readily recognized by the peptidases^20^. Thus, in this study, ^67^Ga-DOTA-(D-Asp)_n_ (Fig. 1B) of varying peptide lengths (n = 2, 5, 8, 11, or 14) were synthesized and evaluated. Moreover, to compare the different acidic amino acids as components of the carrier, ^67^Ga-DOTA-(L-Glu)_14_ (Fig. 1C) and ^67^Ga-DOTA-(D-Glu)_14_ (Fig. 1D) were synthesized and evaluated *in vitro* and *in vivo*.

## Results

### Preparation of ^67^Ga-DOTA-(D-Asp)_n_ (n = 2, 5, 8, 11, or 14), ^67^Ga-DOTA-(L-Glu)_14_, and ^67^Ga-DOTA-(D-Glu)_14_

^67^Ga-DOTA-(D-Asp)_n_ (n = 2, 5, 8, 11, or 14), ^67^Ga-DOTA-(L-Glu)_14_, and ^67^Ga-DOTA-(D-Glu)_14_ were prepared by complexing DOTA-(D-Asp)_n_, DOTA-(L-Glu)_14_, and DOTA-(D-Glu)_14_ with ^67^Ga, respectively. Radiochemical yields of ^67^Ga-DOTA-(D-Asp)_2_, ^67^Ga-DOTA-(D-Asp)_5_, ^67^Ga-DOTA-(D-Asp)_8_, ^67^Ga-DOTA-(D-Asp)_11_, ^67^Ga-DOTA-(D-Asp)_14_, ^67^Ga-DOTA-(L-Glu)_14_, and ^67^Ga-DOTA-(D-Glu)_14_ were 25%, 67%, 74%, 56%, 51%, 38%, and 68% respectively. After RP-HPLC purification, ^67^Ga-DOTA-(D-Asp)_n_, ^67^Ga-DOTA-(L-Glu)_14_, and ^67^Ga-DOTA-(D-Glu)_14_ had radiochemical purities of over 95%. The formation of ^67^Ga-DOTA-(D-Asp)_n_, ^67^Ga-DOTA-(L-Glu)_14_, and ^67^Ga-DOTA-(D-Glu)_14_ complexes were determined by examining the retention times in RP-HPLC analyses. The ^67^Ga-labeled complexes showed identical retention times as the corresponding nonradioactive complexes. The results indicated that the formation of ^67^Ga-labeled complexes were identical to those of nonradioactive Ga complexes, which were determined by MS.

### Hydroxyapatite-binding assay

**Figure 2:**
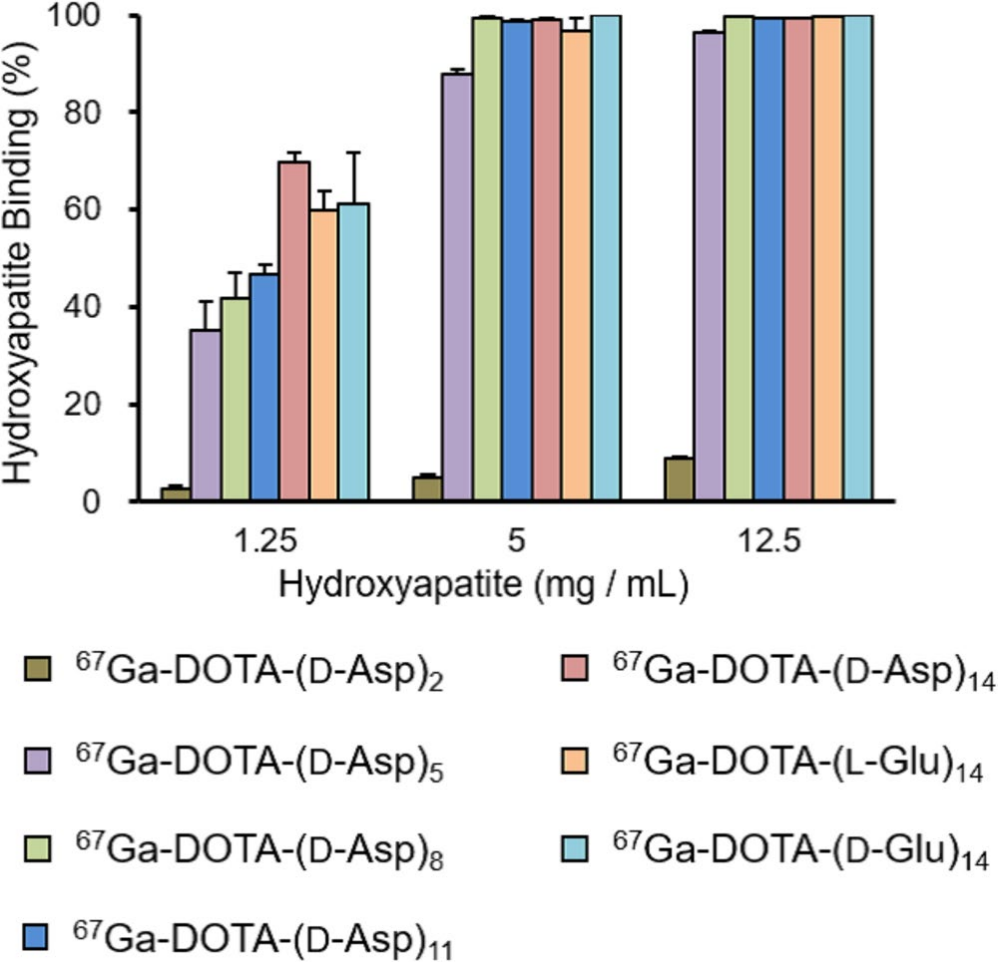
Binding ratios of ^67^Ga-DOTA-(D-Asp)_n_ (n = 2, 5, 8, 11, or 14), ^67^Ga-DOTA-(L-Glu)_14_, and ^67^Ga-DOTA-(D-Glu)_14_ to hydroxyapatite beads. Data are shown as the mean ± SD for four samples.

Figure 2 shows the percentage of each ^67^Ga-DOTA-(D-Asp)_n_ (n = 2, 5, 8, 11, or 14), ^67^Ga-DOTA-(L-Glu)_14_, and ^67^Ga-DOTA-(D-Glu)_14_ bound to hydroxyapatite beads. Binding of each ^67^Ga-DOTA-(D-Asp)_n_ to the beads increased with an increasing amount of hydroxyapatite, except for that of ^67^Ga-DOTA-(D-Asp)_2_. Binding of ^67^Ga-DOTA-(D-Asp)_n_ to hydroxyapatite tended to increase with increasing length of amino acid chain. The binding affinities of ^67^Ga-DOTA-(L-Glu)_14_ and ^67^Ga-DOTA-(D-Glu)_14_ were comparable to that of ^67^Ga-DOTA-(D-Asp)_14_.

### Biodistribution experiments

The biodistribution of ^67^Ga-DOTA-(D-Asp)_n_ (n = 2, 5, 8, 11, or 14), ^67^Ga-DOTA-(L-Glu)_14_, ^67^Ga-DOTA-(D-Glu)_14_, [^18^F]NaF, and ^99m^Tc-MDP in normal mice is shown in Tables 1–9. Among these compounds, ^67^Ga-DOTA-(D-Asp)_8_, ^67^Ga-DOTA-(D-Asp)_11_, ^67^Ga-DOTA-(D-Asp)_14_, ^67^Ga-DOTA-(L-Glu)_14_, ^67^Ga-DOTA-(D-Glu)_14_, [^18^F]NaF, and ^99m^Tc-MDP showed high accumulation and retention of radioactivity in bone. ^67^Ga-DOTA-(D-Asp)_5_ showed moderate accumulation of radioactivity in bone; however, the level of radioactivity decreased 3 h after injection. ^67^Ga-DOTA-(D-Asp)_2_ caused subtle accumulation of radioactivity in bone. Although there was little radioactivity in other tissues at 3 h after the injection of ^67^Ga-DOTA-(D-Asp)_n_, ^99m^Tc-MDP, and [^18^F]NaF because of rapid excretion via the kidneys, the radioactivity in the kidneys after the injection of ^67^Ga-DOTA-(L-Glu)_14_ and ^67^Ga-DOTA-(D-Glu)_14_ was retained.

**Table 1:** Biodistribution of radioactivity after i.v. injection of ^67^Ga-DOTA-(D-Asp)_2_ in mice^a^.

Tissue	Time after injection		
	10 min	60 min	180 min
Blood	2.19 (0.24)	0.24 (0.12)	0.05 (0.03)
Liver	0.57 (0.08)	0.31 (0.27)	0.11 (0.04)
Kidney	7.61 (1.17)	6.33 (4.68)	1.42 (0.59)
Small-intestine	0.50 (0.11)	0.14 (0.08)	0.08 (0.03)
Large-intestine	0.37 (0.08)	0.12 (0.11)	0.38 (0.23)
Spleen	0.50 (0.11)	0.24 (0.14)	0.08 (0.02)
Pancreas	0.57 (0.12)	0.15 (0.06)	0.09 (0.05)
Lung	1.45 (0.23)	0.26 (0.18)	0.08 (0.01)
Heart	0.86 (0.11)	0.11 (0.04)	0.06 (0.03)
Stomach^b^	0.24 (0.07)	0.06 (0.03)	0.36 (0.58)
Bone (Femur)	1.57 (0.71)	0.88 (0.34)	0.49 (0.04)
Muscle	0.53 (0.05)	0.11 (0.05)	0.08 (0.03)
Brain	0.07 (0.01)	0.02 (0.01)	0.01 (0.01)
F/B ratio^c^	0.71 (0.27)	3.78 (1.03)	12.80 (8.77)

^a^Expressed as % injected dose. Each value represents the mean (SD) for five animals.

^b^Expressed as % injected dose.

^c^Femur:blood ratio.

**Table 2:** Biodistribution of radioactivity after i.v. injection of ^67^Ga-DOTA-(D-Asp)_5_ in mice^a^.

Tissue	Time after injection		
	10 min	60 min	180 min
Blood	2.81 (0.73)	0.27 (0.02)	0.13 (0.07)
Liver	0.67 (0.13)	0.22 (0.11)	0.14 (0.03)
Kidney	12.04 (4.05)	6.31 (3.64)	1.45 (0.34)
Small-intestine	0.55 (0.16)	0.23 (0.09)	0.18 (0.19)
Large-intestine	0.47 (0.13)	0.22 (0.27)	0.22 (0.13)
Spleen	0.56 (0.12)	0.16 (0.05)	0.11 (0.03)
Pancreas	0.72 (0.11)	0.25 (0.19)	0.06 (0.00)
Lung	2.03 (0.34)	0.30 (0.11)	0.09 (0.02)
Heart	1.03 (0.29)	0.12 (0.01)	0.06 (0.01)
Stomach^b^	0.32 (0.06)	0.10 (0.06)	0.48 (0.89)
Bone (Femur)	6.78 (1.84)	5.01 (0.93)	2.80 (0.58)
Muscle	0.68 (0.21)	0.13 (0.03)	0.07 (0.03)
Brain	0.10 (0.05)	0.02 (0.01)	0.01 (0.00)
F/B ratio^c^	2.41 (0.22)	18.52 (2.59)	25.42 (10.06)

^a^Expressed as % injected dose. Each value represents the mean (SD) for four animals.

^b^Expressed as % injected dose.

^c^Femur:blood ratio.

**Table 3:** Biodistribution of radioactivity after i.v. injection of ^67^Ga-DOTA-(D-Asp)_8_ in mice^a^.

Tissue	Time after injection		
	10 min	60 min	180 min
Blood	1.94 (0.12)	0.33 (0.05)	0.13 (0.05)
Liver	0.46 (0.06)	0.14 (0.02)	0.11 (0.01)
Kidney	12.79 (9.18)	2.49 (2.15)	1.23 (0.48)
Small-intestine	0.39 (0.06)	0.13 (0.03)	0.10 (0.06)
Large-intestine	0.26 (0.02)	0.05 (0.00)	0.08 (0.01)
Spleen	0.42 (0.02)	0.16 (0.05)	0.09 (0.02)
Pancreas	0.57 (0.06)	0.11 (0.01)	0.08 (0.02)
Lung	1.34 (0.10)	0.26 (0.03)	0.10 (0.03)
Heart	0.63 (0.06)	0.14 (0.07)	0.05 (0.02)
Stomach^b^	0.34 (0.23)	0.04 (0.01)	0.10 (0.13)
Bone (Femur)	9.86 (1.90)	11.63 (2.57)	12.49 (2.61)
Muscle	0.57 (0.40)	0.15 (0.07)	0.14 (0.16)
Brain	0.05 (0.00)	0.01 (0.01)	0.01 (0.00)
F/B ratio^c^	5.04 (0.77)	35.96 (13.10)	122.09 (89.13)

^a^Expressed as % injected dose. Each value represents the mean (SD) for five animals.

^b^Expressed as % injected dose.

^c^Femur:blood ratio.

**Table 4:** Biodistribution of radioactivity after i.v. injection of ^67^Ga-DOTA-(D-Asp)_11_ in mice^a^.

Tissue	Time after injection		
	10 min	60 min	180 min
Blood	1.71 (0.21)	0.14 (0.10)	0.08 (0.04)
Liver	0.51 (0.08)	0.20 (0.10)	0.13 (0.06)
Kidney	12.83 (5.61)	2.92 (3.25)	1.00 (0.40)
Small-intestine	0.38 (0.07)	0.07 (0.01)	0.05 (0.01)
Large-intestine	0.28 (0.02)	0.03 (0.01)	0.11 (0.03)
Spleen	0.44 (0.08)	0.13 (0.06)	0.10 (0.02)
Pancreas	0.53 (0.07)	0.09 (0.09)	0.04 (0.01)
Lung	1.24 (0.18)	0.08 (0.01)	0.05 (0.01)
Heart	0.65 (0.11)	0.04 (0.01)	0.03 (0.01)
Stomach^b^	0.18 (0.03)	0.04 (0.02)	0.02 (0.01)
Bone (Femur)	11.93 (0.55)	15.26 (1.08)	15.45 (1.65)
Muscle	0.58 (0.16)	0.18 (0.10)	0.06 (0.03)
Brain	0.05 (0.01)	0.01 (0.01)	0.02 (0.01)
F/B ratio^c^	7.01 (0.56)	147.10 (71.25)	248.95 (170.64)

^a^Expressed as % injected dose. Each value represents the mean (SD) for five animals.

^b^Expressed as % injected dose.

^c^Femur:blood ratio.

**Table 5:** Biodistribution of radioactivity after i.v. injection of ^67^Ga-DOTA-(D-Asp)_14_ in mice^a^.

Tissue	Time after injection		
	10 min	60 min	180 min
Blood	2.46 (0.60)	0.09 (0.04)	0.03 (0.01)
Liver	0.51 (0.12)	0.15 (0.04)	0.05 (0.03)
Kidney	8.15 (3.86)	1.21 (0.57)	0.54 (0.12)
Small-intestine	0.55 (0.06)	0.18 (0.04)	0.12 (0.02)
Large-intestine	0.40 (0.06)	0.10 (0.01)	0.20 (0.01)
Spleen	0.48 (0.13)	0.12 (0.03)	0.12 (0.04)
Pancreas	0.88 (0.07)	0.27 (0.04)	0.14 (0.06)
Lung	1.75 (0.47)	0.28 (0.09)	0.03 (0.01)
Heart	0.94 (0.16)	0.18 (0.04)	0.11 (0.03)
Stomach^b^	0.28 (0.08)	0.07 (0.01)	0.06 (0.01)
Bone (Femur)	11.90 (2.99)	13.03 (0.90)	14.78 (2.34)
Muscle	0.77 (0.15)	0.16 (0.03)	0.16 (0.11)
Brain	0.06 (0.01)	0.01 (0.01)	0.01 (0.00)
F/B ratio^c^	4.90 (0.70)	180.99 (91.94)	526.37 (130.49)

^a^Expressed as % injected dose. Each value represents the mean (SD) for five animals.

^b^Expressed as % injected dose.

^c^Femur:blood ratio.

**Table 6:** Biodistribution of radioactivity after i.v. injection of ^67^Ga-DOTA-(L-Glu)_14_ in mice^a^.

Tissue	Time after injection		
	10 min	60 min	180 min
Blood	1.85 (0.47)	0.12 (0.04)	0.05 (0.02)
Liver	0.47 (0.16)	0.13 (0.03)	0.14 (0.04)
Kidney	22.06 (4.67)	26.41 (8.53)	25.14 (3.75)
Small-intestine	0.50 (0.14)	0.13 (0.05)	0.15 (0.09)
Large-intestine	0.37 (0.13)	0.07 (0.01)	0.23 (0.15)
Spleen	0.44 (0.12)	0.10 (0.03)	0.11 (0.06)
Pancreas	0.59 (0.15)	0.13 (0.02)	0.08 (0.02)
Lung	1.57 (0.42)	0.13 (0.03)	0.06 (0.02)
Heart	0.67 (0.22)	0.10 (0.02)	0.06 (0.01)
Stomach^b^	0.22 (0.03)	0.07 (0.05)	0.07 (0.07)
Bone (Femur)	9.81 (2.35)	11.07 (1.66)	10.90 (1.17)
Muscle	0.63 (0.12)	0.10 (0.05)	0.30 (0.47)
Brain	0.05 (0.01)	0.01 (0.00)	0.03 (0.01)
F/B ratio^c^	5.41 (0.93)	97.50 (26.27)	239.85 (89.95)

^a^Expressed as % injected dose. Each value represents the mean (SD) for five animals.

^b^Expressed as % injected dose.

^c^Femur:blood ratio.

**Table 7:** Biodistribution of radioactivity after i.v. injection of ^67^Ga-DOTA-(D-Glu)_14_ in mice^a^.

Tissue	Time after injection		
	10 min	60 min	180 min
Blood	2.09 (0.35)	0.10 (0.02)	0.01 (0.01)
Liver	0.46 (0.07)	0.12 (0.03)	0.08 (0.02)
Kidney	13.43 (3.15)	10.61 (5.38)	6.59 (1.74)
Small-intestine	0.37 (0.03)	0.22 (0.15)	0.15 (0.05)
Large-intestine	0.42 (0.05)	0.18 (0.17)	0.25 (0.12)
Spleen	0.37 (0.06)	0.06 (0.02)	0.03 (0.02)
Pancreas	0.68 (0.26)	0.13 (0.05)	0.06 (0.02)
Lung	1.65 (0.44)	0.13 (0.02)	0.04 (0.01)
Heart	0.79 (0.13)	0.08 (0.02)	0.05 (0.02)
Stomach^b^	0.29 (0.06)	0.19 (0.11)	0.29 (0.23)
Bone (Femur)	10.98 (0.49)	11.78 (1.21)	12.20 (2.41)
Muscle	0.78 (0.31)	0.19 (0.22)	0.02 (0.01)
Brain	0.05 (0.01)	0.01 (0.00)	0.01 (0.00)
F/B ratio^c^	5.39 (1.05)	129.55 (40.09)	1179.73 (699.19)

^a^Expressed as % injected dose. Each value represents the mean (SD) for four animals.

^b^Expressed as % injected dose.

^c^Femur:blood ratio.

**Table 8:** Biodistribution of radioactivity after i.v. injection of [^18^F]NaF in mice^a^.

Tissue	Time after injection		
	10 min	60 min	180 min
Blood	1.78 (0.23)	0.11 (0.04)	0.02 (0.00)
Liver	1.34 (0.17)	0.09 (0.02)	0.02 (0.00)
Kidney	5.23 (2.31)	1.13 (0.89)	0.15 (0.08)
Small-intestine	1.31 (0.22)	0.68 (0.10)	0.06 (0.02)
Large-intestine	0.95 (0.22)	1.21 (0.27)	1.39 (0.19)
Spleen	1.10 (0.15)	0.08 (0.02)	0.02 (0.00)
Pancreas	0.87 (0.15)	0.08 (0.08)	0.02 (0.03)
Lung	1.44 (0.19)	0.10 (0.03)	0.03 (0.01)
Heart	1.84 (0.41)	0.15 (0.05)	0.02 (0.01)
Stomach^b^	0.36 (0.04)	0.06 (0.02)	0.13 (0.14)
Bone (Femur)	27.69 (3.15)	39.96 (2.52)	43.91 (2.64)
Muscle	0.83 (0.13)	0.07 (0.04)	0.01 (0.01)
Brain	0.10 (0.02)	0.36 (0.52)	0.05 (0.02)
F/B ratio^c^	15.59 (1.01)	381.63 (84.17)	1983.88 (256.88)

^a^Expressed as % injected dose. Each value represents the mean (SD) for four animals.

^b^Expressed as % injected dose.

^c^Femur:blood ratio.

**Table 9:** Biodistribution of radioactivity after i.v. injection of ^99m^Tc-MDP in mice^a^.

Tissue	Time after injection		
	10 min	60 min	180 min
Blood	2.43 (0.10)	0.23 (0.04)	0.05 (0.01)
Liver	0.61 (0.02)	0.27 (0.05)	0.16 (0.04)
Kidney	10.44 (1.56)	2.03 (0.50)	1.22 (0.36)
Small-intestine	0.69 (0.15)	1.55 (1.68)	0.26 (0.08)
Large-intestine	0.61 (0.20)	0.14 (0.04)	0.23 (0.07)
Spleen	0.54 (0.07)	0.14 (0.02)	0.07 (0.01)
Pancreas	0.77 (0.10)	0.13 (0.02)	0.07 (0.01)
Lung	1.80 (0.16)	0.30 (0.03)	0.10 (0.02)
Heart	0.94 (0.05)	0.16 (0.02)	0.07 (0.02)
Stomach^b^	0.63 (0.14)	0.70 (0.37)	0.29 (0.07)
Bone (Femur)	20.76 (1.51)	27.92 (3.25)	29.03 (2.12)
Muscle	0.57 (0.09)	0.13 (0.04)	0.06 (0.01)
Brain	0.06 (0.01)	0.02 (0.00)	0.01 (0.00)
F/B ratio^c^	8.54 (0.57)	120.87 (9.30)	546.64 (91.83)

^a^Expressed as % injected dose. Each value represents the mean (SD) for four animals.

^b^Expressed as % injected dose.

^c^Femur:blood ratio.

### Urine Analyses

The results of urine analysis using RP-HPLC after injection of ^67^Ga-DOTA-(L-Asp)_14_ and ^67^Ga-DOTA-(D-Asp)_14_ showed that a part of these complexes metabolized to more hydrophilic complexes; some radioactivity was eluted earlier than the intact complex. The ratio of the intact complex after injection of ^67^Ga-DOTA-(D-Asp)_14_ (85.8 ± 17.4%) was significantly higher than that of ^67^Ga-DOTA-(L-Asp)_14_ (55.0 ± 13.9%).

## Discussion

It has been shown that the bisphosphonate structure is very useful as a carrier of physiologically active molecules or compounds with medicinal properties. This is particularly true for bone lesions because of the high affinity of bisphosphonate for hydroxyapatite, which is plentiful in bone but not in soft tissues^21,22^. Stable radiometal complex-conjugated bisphosphonate compounds have been designed as bone-seeking radiopharmaceuticals; they have been synthesized and evaluated for the diagnosis and therapy of bone metastases^11,23–30^. The available data show that bisphosphonate is an excellent carrier of radioisotopes to bone lesions. Our recent study has shown that L-aspartic acid peptides could also work as carriers of radioisotopes to bone lesions; L-aspartic acid peptides have high affinity for hydroxyapatite^19,31^. Thus, we assumed that D-aspartic acid peptides might be even better carriers. They should have a similar degree of affinity for hydroxyapatite but higher stability *in vivo* than the L-aspartic acid compounds.

In the hydroxyapatite-binding assay, ^67^Ga-DOTA-(D-Asp)_n_ with a longer amino acid chain showed higher affinity for hydroxyapatite than the short-chain compounds. The binding patterns of ^67^Ga-DOTA-(D-Asp)_n_ were almost the same as those of ^67^Ga-DOTA-(L-Asp)_n_^19^. A previous study reported that the dissociation constants and the maximal binding rates of Fmoc-peptide compounds for hydroxyapatite show no significant differences among Fmoc-(L-Asp)_n_, Fmoc-(D-Asp)_n_, and Fmoc-(L-Glu)_n_ (n = 2, 4, 6, 8, 10)^32^. This is consistent with the results of hydroxyapatite binding assay in our study. We found that aspartic acid peptides had the same degree of affinity for hydroxyapatite regardless of their optical isomeric form. Moreover, there were no differences between the affinities of aspartic acid peptides and glutamic acid peptides for hydroxyapatite.

In *in vivo* studies, it is known that the peptides that composed of D-amino acids are more stable than the L-amino acid peptides^20^. A study examining the Fmoc compounds reported that, after a single i.v. administration, the plasma concentration of Fmoc-(L-Asp)_6_ decreased more rapidly than the concentration of Fmoc-(D-Asp)_6_. Degradation products did not appear in the plasma after the injection of Fmoc-(D-Asp)_6_, but Fmoc-(L-Asp)_4_ and Fmoc-(L-Asp)_2_ were detected in plasma after the injection of Fmoc-(L-Asp)_6_^32^. Therefore, we had expected to observe increased accumulation in bone after the injection of ^67^Ga-DOTA-(D-Asp)_n_, caused by their superior *in vivo* stability. In urine analyses, ^67^Ga-DOTA-(L-Asp)_14_ metabolized to more hydrophilic complexes, which should be ^67^Ga-DOTA conjugated with shorter aspartic acid peptides, because of the cleavage of an amide bond in the peptide. These compounds were diluted before the full-length compound during the RP-HPLC using an ODS column. This indicates that ^67^Ga-DOTA-(D-Asp)_14_ is more stable than ^67^Ga-DOTA-(L-Asp)_14_. Since ^67^Ga-DOTA conjugated with shorter aspartic acid peptides should show lower accumulation in bone than ^67^Ga-DOTA conjugated with long aspartic acid peptides, we expected that ^67^Ga-DOTA-(D-Asp)_n_, which has higher stability, would show higher accumulation in bone than ^67^Ga-DOTA-(L-Asp)_n_. However, against our expectations, the accumulation of radioactivity in bone was comparable for ^67^Ga-DOTA-(L-Asp)_n_ and ^67^Ga-DOTA-(D-Asp)_n_. Not only ^67^Ga-DOTA-(D-Asp)_n_ but also ^67^Ga-DOTA-(L-Asp)_n_ immediately accumulated in bone or was excreted into urine via the kidneys with little degradation; both molecule types showed extremely rapid clearance from the blood. There was no difference between the biodistributions of ^67^Ga-DOTA-(L-Asp)_n_ and ^67^Ga-DOTA-(D-Asp)_n_.

To compare the biodistributions of ^67^Ga-DOTA-conjugated acidic amino acid peptides with the biodistributions of other typical bone-seeking compounds, biodistribution experiments of ^99m^Tc-MDP and [^18^F]NaF were performed. ^67^Ga-DOTA-(D-Asp)_11_, ^67^Ga-DOTA-(D-Asp)_14_, ^99m^Tc-MDP, and [^18^F]NaF showed excellent biodistribution as bone imaging agents, such as high bone accumulation and low radioactivity in non-target tissues. Among these agents, as [^18^F]NaF showed the highest bone uptake, [^18^F]NaF may have the most preferable biodistribution as a bone imaging agent. However, the bone/non-target tissue radioactivity ratios of ^99m^Tc-MDP and ^67^Ga-DOTA-(D-Asp)_n_ (n = 11 or 14) are sufficient for bone imaging, and ^99m^Tc and ^68^Ga have some convenient physical properties as radionuclides. Thus, ^99m^Tc-MDP and ^68^Ga-DOTA-(D-Asp)_n_ (n = 11 or 14) should be useful in a clinical context.

The ^67^Ga-DOTA-conjugated L-glutamic acid peptide, ^67^Ga-DOTA-(L-Glu)_14_, and the ^67^Ga-DOTA-conjugated D-glutamic acid peptide, ^67^Ga-DOTA-(D-Glu)_14_, also showed rapid clearance from the blood and high accumulation in bone, similarly to ^67^Ga-DOTA-(L-Asp)_14_ and ^67^Ga-DOTA-(D-Asp)_14_. Generally, radiometal-labeled peptides tend to show a high accumulation of radioactivity in the kidneys. It has been reported that the accumulation of radioactivity in the kidneys after the injection of ^111^In-labeled peptides is affected by their molecular charges^33,34^. As the renal brush border membrane is negatively charged, a repulsive force could arise between this membrane and negatively charged compounds. Such repulsive force could inhibit the reabsorption of these compounds into renal proximal tubular cells. The introduction of negative charges into radiometal-labeled peptides has also been studied to develop a method of decreasing the accumulation of radioactivity in the kidneys^35^. The extremely low accumulation of radioactivity in the kidneys after the injection of ^67^Ga-DOTA-(L-Asp)_14_ and ^67^Ga-DOTA-(D-Asp)_14_ may have been caused by their negative charges. We had expected that ^67^Ga-DOTA-(L-Glu)_14_ and ^67^Ga-DOTA-(D-Glu)_14_, being negatively charged like ^67^Ga-DOTA-(L-Asp)_14_ and ^67^Ga-DOTA-(D-Asp)_14_, would also cause low accumulation of radioactivity in the kidneys. However, contrary to our expectations, high accumulation and retention or slower clearance of radioactivity in the kidneys were observed after the injection of ^67^Ga-DOTA-(L-Glu)_14_ or ^67^Ga-DOTA-(D-Glu)_14_. The mechanism behind these phenomena are unclear, but we must conclude that the glutamic acid peptides are not appropriate as carriers to the bone in the nuclear medicine imaging because of their association with high radioactivity in the kidneys.

In this study, no differences in the biodistributions between L-aspartic acid [^67^Ga-DOTA-(L-Asp)_n_] and D-aspartic acid [^67^Ga-DOTA-(D-Asp)_n_] compounds were observed, presumably because of their extremely rapid blood clearance. Recently, we have proposed a new concept of using a bifunctional peptide containing an aspartic acid peptide linker as a carrier to bone metastases and an RGD peptide, which has high affinity for α_v_β_3_ integrin, as a carrier to primary cancer^31^. In this compound, L-aspartic acid is used as a composite component of the aspartic acid peptide linker. A D-aspartic acid peptide linker may be effective in the new approach. Higher stability of the D-aspartic acid peptide linker should be effective for higher accumulation in target tissues because the blood clearance of bifunctional peptide does not occur as rapidly as that of ^67^Ga-DOTA-(D-Asp)_n_. Further studies are needed to examine the effectiveness of a D-aspartic acid peptide linker in the drug design concept.

## Methods

### Materials

Electrospray ionization mass (ESI-MS) analyses were performed with a LCQ (Thermo Fisher Scientific, Waltham, MA, USA). Matrix assisted laser desorption/ionization-time of flight mass (MALDI-TOF-MS) analyses were performed with ABI 4800 plus (AB SCIEX, Foster, CA, USA). [^67^Ga]GaCl_3_ was supplied by Nihon Medi-Physics Co., Ltd. (Tokyo, Japan). [^18^F]NaF was prepared in Fukui University and transported to Kanazawa University. [^99m^Tc]Pertechnetate (^99m^TcO_4_^−^) was eluted in saline solution from generators (Nihon Medi-Physics Co., Ltd). ^99m^Tc-MDP was prepared by the addition of ^99m^TcO_4_^−^ solution into the mixture of MDP (Wako Pure Chemical Industries, Ltd., Osaka, Japan), tin(II) chloride, and ascorbic acid solution. 1,4,7,10-Tetraazacyclododecane-1,4,7-tris(t-butyl acetate) (DOTA-tris) was purchased from Macrocyclics (Dallas, TX, USA). 9-Fluorenylmethoxycarbonyl (Fmoc)-D-Asp(OtBu)-Wang resin, Fmoc-D-Asp(OtBu), and Fmoc-L-Glu(OtBu) were purchased from Merck KGaA (Darmstadt, Germany). Fmoc-L-Glu(OtBu)-Wang resin and 2-chlorotrityl chloride resin were purchased from Watanabe chemical Industries, LTD. (Hiroshima, Japan). Fmoc-D-Glu(OtBu) was purchased from Tokyo Chemical Industry Co., Ltd. (Tokyo, Japan). Other reagents were of reagent grade and used as received.

### Synthesis of DOTA-(D-Asp)_n_ (n = 2, 5, 8, 11, or 14)

The protected peptidyl resin was manually constructed by an Fmoc-based solid-phase methodology using a method described previously^19^. After the construction of the peptide chain on the resin, the Fmoc protecting group was removed using 20% piperidine in dimethylformamide (DMF), and a mixture containing two equivalents of DOTA-tris, 1,3-diisopropylcarbodiimide (DIPCDI), and 1-hydroxybenzotriazole hydrate (HOBt) in dimethylformamide (DMF) was added and allowed to react for 2 h. For the cleavage of peptides from the resin and deprotection, 0.5 mL of thioanisole and 5 mL of trifluoroacetic acid (TFA) were added to the completely protected peptide resin at 0 °C. After stirring at room temperature for 2 h, the resin was removed by filtration, and ether was added to the filtrate at 0 °C to precipitate crude peptide. The crude products were purified by reversed-phase (RP)-HPLC using a Hydrosphere 5C18 column (10 × 150 mm; YMC, Kyoto, Japan) at a flow rate of 4 mL/min with an isocratic mobile phase of water containing 0.1% TFA [in the case of DOTA-(D-Asp)_2_] or using a Cosmosil 5C_18_-AR 300 column (10 × 150 mm; Nacalai Tesque, Kyoto, Japan) at a flow rate of 4 mL/min with a gradient mobile phase from water containing 0.1% TFA to 20% methanol in water containing 0.1% TFA for 20 min [in the case of DOTA-(D-Asp)_n_ (n = 5, 8, 11, or 14)]. UV Chromatograms (220 nm) were obtained. The fraction containing DOTA-(D-Asp)_n_ (n = 2, 5, 8, 11, or 14) was determined by mass spectrometry and collected. The solvent removal from the fraction was performed by freeze-drying to provide DOTA-(D-Asp)_n_ as white powder.

DOTA-(D-Asp)_2_ MS (ESI): *m/z* 635 (M + H)^+^, Yield: 30.4%

DOTA-(D-Asp)_5_ MS (ESI): *m/z* 980 (M + H)^+^, Yield: 39.8%

DOTA-(D-Asp)_8_ MS (ESI): *m/z* 1325 (M + H)^+^, Yield: 11.7%

DOTA-(D-Asp)_11_ MS (ESI): *m/z* 1670 (M + H)^+^, Yield: 12.1%

DOTA-(D-Asp)_14_ MS (MALDI): *m/z* 2015 (M + H)^+^, Yield: 13.6%

### Synthesis of DOTA-(L-Glu)_14_

A resin-binding protected peptide was constructed by same procedure as mentioned above using Fmoc-L-Glu(OtBu)-Wang resin, Fmoc-L-Glu(OtBu), and tris-DOTA. For the cleavage of peptides from the resin and the deprotection, 0.5 mL of thioanisole and 5 mL of TFA were added to the fully protected peptide resin at 0 °C. After stirring at room temperature for 2 h, the crude product was purified by RP-HPLC at a flow rate of 4 mL/min with a gradient mobile phase from water containing 0.1% TFA to 20% methanol in water containing 0.1% TFA for 20 min. The solvent removal from the fraction was performed by freeze-drying to provide DOTA-(L-Glu)_14_ and as white powder.

DOTA-(L-Glu)_14_ MS (ESI): *m/z* 2212 (M + H)^+^, Yield: 14.6%

### Synthesis of DOTA-(D-Glu)_14_

Fmoc-D-Glu(OtBu) (4 molar equivalents to resin) was dissolved in dichloromethane. 2-Chlorotrityl chloride resin and *N*,*N*-diisopropylethylamine (DIEA, 3.5 equiv.) were added. The reaction mixture was rotated for 1 h, and 1 mL of methanol was added to react further for 30 min at room temperature. Construction, cleavage, deprotection, and purification of the peptide were performed by the same procedure as mentioned above. DOTA-(D-Glu)_14_ was obtained as white powder.

DOTA-(D-Glu)_14_ MS (ESI): *m/z* 2212 (M + H)^+^, Yield: 2.1%

### Preparation of Ga-DOTA-(D-Asp)_n_ (n = 2, 5, 8, 11, or 14), Ga-DOTA-(L-Glu)_14_, and Ga-DOTA-(D-Glu)_14_

Ga-DOTA-(D-Asp)_n_ (n = 2, 5, 8, 11, or 14), Ga-DOTA-(L-Glu)_14_, and Ga-DOTA-(D-Glu)_14_ were synthesized using a method described previously^19^.

Ga-DOTA-(D-Asp)_2_ MS (ESI): *m/z* 701 (M)^+^

Ga-DOTA-(D-Asp)_5_ MS (ESI): *m/z* 1046 (M)^+^

Ga-DOTA-(D-Asp)_8_ MS (ESI): *m/z* 1391 (M)^+^

Ga-DOTA-(D-Asp)_11_ MS (ESI): *m/z* 1736 (M)^+^

Ga-DOTA-(D-Asp)_14_ MS (MALDI): *m/z* 2081 (M)^+^

Ga-DOTA-(L-Glu)_14_ MS (ESI): *m/z* 2278 (M)^+^

Ga-DOTA-(D-Glu)_14_ MS (ESI): *m/z* 2278 (M)^+^

### Preparation of ^67^Ga-DOTA-(D-Asp)_n_ (n = 2, 5, 8, 11, or 14), ^67^Ga-DOTA-(L-Glu)_14_, and ^67^Ga-DOTA-(D-Glu)_14_

Approximately 50 μg of DOTA-(D-Asp)_n_ (n = 2, 5, 8, 11, or 14), DOTA-(L-Glu)_14_ or DOTA-(D-Glu)_14_ was dissolved in 75 μL of 0.2 M ammonium acetate buffer (pH 5.0), and 25 μL of ^67^GaCl_3_ solution (1.85 MBq) in 0.01 M HCl was added and allowed to react at 80 °C for 8 min. ^67^Ga-labeled peptides were purified by RP-HPLC performed using a Hydrosphere 5C18 column (4.6 × 250 mm; YMC) at a flow rate of 1 mL/min with an isocratic mobile phase of water containing 0.1% TFA [in the case of ^67^Ga-DOTA-(D-Asp)_2_] or using a Cosmosil 5C_18_-AR 300 column (4.6 × 150 mm) at a flow rate of 1 mL/min with a gradient mobile phase from water containing 0.1% TFA to 20% methanol in water containing 0.1% TFA for 20 min [in the case of ^67^Ga-DOTA-(D-Asp)_n_ (n = 5, 8, 11, or 14), ^67^Ga-DOTA-(L-Glu)_14_, and ^67^Ga-DOTA-(D-Glu)_14_].

### Preparation of [^18^F]NaF

No-carrier-added [^18^F]fluoride was produced via the ^18^O(p,n)^18^F reaction from > 98% enriched [^18^O]water (Taiyo Nippon Sanso Corporation, Tokyo, Japan) on an RDS eclipse RD/HP medical cyclotron (Siemens, Knoxville, TN, USA). [^18^F]NaF was prepared by eluting [^18^F]fluoride trapped on an anion exchange column (QMA Plus Light; Waters Corporation, Milford, MA, USA) with saline after washing the anion exchange column with water.

### Hydroxyapatite-binding assays

Hydroxyapatite-binding assays were performed in accordance with previously described procedures^19,25^. In brief, hydroxyapatite beads (Bio-Gel; Bio-Rad, Hercules, CA, USA) were suspended in Tris/HCl-buffered saline (50 mM, pH 7.4) at 2.5 mg/mL, 10 mg/mL, and 25 mg/mL. For the solutions of ^67^Ga-DOTA-(D-Asp)_n_ (n = 2, 5, 8, 11, or 14), ^67^Ga-DOTA-(L-Glu)_14_, or ^67^Ga-DOTA-(D-Glu)_14_, ligand concentrations were adjusted to 19.5 µM by adding DOTA-(D-Asp)_n_, DOTA-(L-Glu)_14_, or DOTA-(D-Glu)_14_. Two hundred microliters of each of ^67^Ga-DOTA-(D-Asp)_n_, ^67^Ga-DOTA-(L-Glu)_14_, or ^67^Ga-DOTA-(D-Glu)_14_ solution was added to 200 μL of the hydroxyapatite suspension, and samples were gently shaken for 1 h at room temperature. After centrifugation at 10,000 *g* for 5 min, a part of the radioactivity in the supernatants was measured using a gamma counter (AccuFLEX γ ARC-7010, Hitachi, Ltd., Tokyo, Japan). Control experiments were performed according to the same procedure without hydroxyapatite beads, which showed < 0.1% adsorption of radioactivity to vials. The ratios of binding were determined as follows:

Hydroxyapatite binding (%) = (1 − [sample supernatant radioactivity]/[control supernatant radioactivity]) × 100

### Biodistribution experiments

Experiments with animals were conducted in strict accordance with the Guidelines for the Care and Use of Laboratory Animals of Kanazawa University. The animal experimental protocols used were approved by the Committee on Animal Experimentation of Kanazawa University (Permit Number: AP-132633). Biodistribution experiments were performed after intravenous administration of each diluted tracer solution (37–740 kBq/100 μL) to 6-week-old male ddY mice (27–32 g, Japan SLC, Inc., Hamamatsu, Japan). Four or five mice at each time point after the administration of each compound were sacrificed by decapitation at 10, 60, and 180 min post-injection. Tissues of interest were taken and weighed. Complete left femurs were isolated as representative bone samples, radioactivity was determined using gamma counters (AccuFLEX γ ARC-8001 in the case of ^18^F, Hitachi, Ltd.), and background counts and physical decay were corrected during counting.

### Urine Analyses

^67^Ga-DOTA-(L-Asp)_14_ was prepared according to a method described previously^19^. ^67^Ga-DOTA-(L-Asp)_14_ or ^67^Ga-DOTA-(D-Asp)_14_ solution (370 kBq / 200 μL) was intravenously injected to 6-week-old male ddY mice. At 1 h post-injection, mice were sacrificed and their urea samples were taken from the bladders. After ultrafiltration (Microcon-30, Merck KGaA), the filtrate samples were analyzed by RP-HPLC at a flow rate of 1 mL/min with a gradient mobile phase from water containing 0.1% TFA to 20% methanol in water containing 0.1% TFA for 20 min.
